# Abdominal obesity vs general obesity for identifying arterial stiffness, subclinical atherosclerosis and wave reflection in healthy, diabetics and hypertensive

**DOI:** 10.1186/1471-2261-12-3

**Published:** 2012-02-01

**Authors:** Jose I Recio-Rodriguez, Manuel A Gomez-Marcos, Maria C Patino-Alonso, Cristina Agudo-Conde, Emiliano Rodriguez-Sanchez, Luis Garcia-Ortiz

**Affiliations:** 1 Primary Care Research Unit, La Alamedilla Health Center, SACYL, REDIAPP. Salamanca, Spain; 2 Statistics Department, University of Salamanca, Salamanca, Spain

## Abstract

**Background:**

Our aim was to analyze the relationship between abdominal obesity and general obesity, with subclinical atherosclerosis, arterial stiffness and wave reflection in healthy, diabetics and hypertensive subjects.

**Methods:**

A cross-sectional descriptive study was made of 305 individuals (diabetics 32.8%, hypertensive subjects 37.0% and healthy individuals 30.2%). Measurements: Body mass index (BMI), waist circumference (WC), body fat percentage (BFP) and waist/height ratio (WHtR). Arterial stiffness was assessed according to pulse wave velocity (PWV), intima-media thickness of the common carotid artery (C-IMT), augmentation index (central and peripheral), ankle-brachial index (ABI), and central and peripheral pulse pressure.

**Results:**

WC and WHtR showed a positive correlation to PWV and C-IMT in the studied groups. After adjusting for age, gender, high sensitivity c-reactive protein, serum glucose and the presence of diabetes, hypertension, smoking, dyslipidemia, antidiabetic drugs, lipid-lowering drugs, and atherosclerotic plaques, it was seen that for every 0.1 point increase in WHtR, and for every cm increase in WC, the PWV increased 0.041 and 0.029 m/sec, and C-IMT increased 0.001 mm and 0.001 mm, respectively.

**Conclusions:**

The measures of abdominal obesity (WHtR and WC) correlates better than BMI and BFP with arterial stiffness evaluated by PWV, and with subclinical atherosclerosis evaluated by C-IMT, independently of the presence of diabetes or hypertension.

**Trial Registration:**

Clinical Trials.gov Identifier: NCT01325064

## Background

Obesity is a determinant factor in the development of cardiovascular diseases, and is associated to an increased incidence of hypertension, diabetes, metabolic syndrome and cardiac target organ damage [1-4].

Some studies have shown measures of abdominal obesity such as waist circumference (WC), waist to hip ratio and waist/height ratio (WHtR) to be the parameters best correlated with cardiovascular disease and mortality [5-13]. In contrast, other studies have not found sufficient evidence that these measures of abdominal obesity are superior to body mass index (BMI) in predicting cardiovascular and cardiometabolic risk [14-21].

The vascular structure and function can be assessed through the indices of subclinical atherosclerosis, arterial stiffness and wave reflection [22]. A relationship has been found between measures that assess excess body weight or obesity to certain parameters that measure arterial stiffness and subclinical atherosclerosis, such as the pulse wave velocity (PWV) and the intima-media thickness of the common carotid artery (C-IMT), though their correlation to the augmentation index is not clear [23-25]. However, to our knowledge, no studies have examined whether this relationship differs in healthy subjects, diabetics and hypertensive individuals.

The present study explores the relationship between anthropometric indices that assess abdominal obesity (WC, WHtR) and general obesity (BMI and body fat percentage (BFP)), with parameters that measure arterial stiffness (PWV, central and peripheral pulse pressure), subclinical atherosclerosis (C-IMT and and ankle-brachial index (ABI)) and wave reflection (central augmentation index) in healthy, diabetics and hypertensive subjects.

## Methods

A cross-sectional study was performed in a primary care setting. We consecutively included all the hypertensive, diabetics and healthy patients, that visited their family doctor, aged 20-75 years, from January 2010 to January 2011. After dealing with the reason for consultation, the patients were referred to the research unit for the assessment of cardiovascular risk. Exclusion criteria were: patients with intermittent claudication, and previous cardiovascular events, patients unable to comply with the protocol requirements (psychological and/or cognitive disorders, failure to cooperate, educational limitations and problems for understanding written language, failure to sign the informed consent document), patients participating or who will participate in a clinical trial during the study. The sample size to detect a minimum correlation coefficient between anthropometric parameters and arterial stiffness parameters of 0.3 in diabetic, hypertensive and healthy subject with two-sided type I error rate of 5% and 80% power was estimated to be 85 individuals each group (total 255). We considered enough with the 305 subjects included in the study. The study was approved by an independent ethics committee of Salamanca University Hospital (Spain) and all participants gave written informed consent according to the general recommendations of the Declaration of Helsinki [26].

### Variables and measurement instruments

#### Anthropometric measurements

Body weight was determined on two occasions using a homologated electronic scale (Seca 770) following due calibration (precision ± 0.1 kg), with the patient wearing light clothing and no shoes. These readings was rounded to 100 g. Height in turn was measured with a portable system (Seca 222), recording the average of two readings, and with the patient shoeless in the standing position. The values was rounded to the closest centimeter. Body mass index (BMI) was calculated as weight (kg) divided by height squared (m^2^). A value higher of 30 Kg/m^2 ^was considered obesity. Waist circumference was measured as following: the upper border of the iliac crests are located, and the tape is wrapped around above this point, parallel to the floor, ensuring that it is adjusted without compressing the skin. The reading is taken at the end of a normal breath according to the recommendations of the 2007 SEEDO Conference [27,28], whereas normal when the value is below 102 cm in men and 88 cm in women. WHtR was calculated as height (cm) divided by waist circumference (cm), whereas normal when the value is below 0.5 [29]. Body fat percentage was measured using a body fat monitor (OMRON, model BF306).

#### Blood pressure

**Office or clinical blood pressure **was measured involving three measurements of systolic and diastolic blood pressure, using the average of the last two measurements, with a validated OMRON model M7 sphygmomanometer (Omron Health Care, Kyoto, Japan). Measures were taken in the right upper arm of participants in a sedentary position while they were seated after having rested for at least 5 min. with an appropriately sized cuff based on the measurement of arm circumference and following the recommendations of the European Society of Hypertension [30].

**Central blood pressure and central and peripheral augmentation index (CAIx, PAIx) **were estimated using the SphygmoCor System. With the patient sitting and the arm resting on a rigid surface, pulse wave in radial artery was tested and used to estimate the aortic pulse wave using a mathematic transformation. Inter-observer reliability was assessed before the start of the study using an intraclass correlation of 0.974 (95% CI: 0.936 to 0.989) in repeated measures in 22 subjects and with Bland-Altman analysis, where inter-observer agreement limits were 0.454 (95% CI: -9.876 to 10.785).

**Pulse wave velocity **(PWV) was estimated with the SphygmoCor System (AtCor Medical Pty Ltd Head Office, West Ryde, Australia), with the patient in the supine position. The pulse wave of the carotid and femoral arteries were analyzed, estimating the delay with respect to the ECG wave and calculating PWV. Distance measurements were taken with a measuring tape from the sternal notch to the carotid and femoral arteries at the sensor location. PWV higher than 12 m/sec was considered abnormal [31].

##### Assessment of carotid intima-media thickness (C-IMT)

Carotid ultrasonography to assess IMT was performed by two investigators trained for this purpose before starting the study. Reliability was evaluated before the study began, using the intraclass correlation coefficient, which showed values of 0.974 (95%CI: 0.935 to 0.990) for intraobserver agreement on repeated measurements in 20 subjects, and 0.897 (95%CI: 0.740 to 0.959) for inter-observer agreement. In turn, according to the Bland-Altman analysis, the limit of inter-observer agreement was 0.022 (95%CI: -0.053 to 0.098) and the limit of intra-observer agreement was 0.012 (95%CI: -0.034 to 0.059). A Sonosite Micromax ultrasound device paired with a 5-10 MHz multifrequency high-resolution linear transducer with Sonocal software was used for performing automatic measurements of IMT, in order to optimize reproducibility. Measurements were made of the common carotid artery after the examination of a longitudinal section of 10 mm at a distance of 1 cm from the bifurcation, performing measurements in the proximal wall, and in the distal wall in the lateral, anterior and posterior projections, following an axis perpendicular to the artery to discriminate two lines - one for the intima-blood interface and the other for the media-adventitia interface. A total of 6 measurements were obtained of the right carotid and another 6 of the left carotid, using average values (average IMT) calculated automatically by the software. The measurements were obtained with the subject lying down, with the head extended and slightly turned opposite to the carotid examined, following the recommendations of the Manheim Carotid Intima-Media Thickness Consensus [32]. The average IMT was considered abnormal if it measured > 0.90 mm, or if there were atherosclerotic plaques with a diameter of 1.5 mm or a focal increase of 0.5 mm or 50% of the adjacent IMT [31].

##### Evaluation of peripheral artery involvement

This was evaluated using the ankle-brachial index (ABI), performed in the morning without having consumed coffee or tobacco for at least 8 hours prior to measuring and an ambient temperature of 22-24°C. With the feet uncovered, in a supine decubitus position after 20 minutes of rest, the pressure in the lower extremities and blood pressure in both arms was measured using a portable WatchBP Office ABI (Microlife AG Swiss Corporation). The ABI was calculated automatically for each foot by dividing the higher of the two systolic pressures in the ankle by the highest measurement of the two systolic pressures in the arm. ABI lower than 0.9 was considered abnormal [31,33].

Blood samples were collected in the morning, after patient fasting 8 h. Basal glucose, HDL cholesterol, LDL cholesterol, total cholesterol, triglycerides and fibrinogen were determined. High sensitivity C-reactive protein was determined by the nephelometric method (Beckman Instrument APS; Beckman Coulter Inc., Fullerton, CA, USA) [34]. The parameters were measured on a blind basis in the Hospital Biochemistry laboratory using standard automatized techniques. The HOMA index was calculated as fasting insulin concentration (μU/mL) × fasting glucose concentration (mmol/L)/22.5.

The individuals performing the different tests were blinded to the clinical data of the patient.

### Statistical analysis

The continuous variables were expressed as mean ± standard deviation, while frequency distribution was used in the qualitative variables. The difference in means in quantitative variables between the three groups has been analyzed using the ANOVA for independent samples. Non-normally distributed variables were evaluated by the Kruskal-Wallis test, and they were log transformed for further analysis. Because of their skewed distribution, glucose, triglyceride, high sensitivity c-reactive protein and HOMA were presented as median and interquartile range. Pearson's or Spearman correlation coefficient were used to estimate the relationship between the quantitative variables, while the chi-square test was used to associate the qualitative variables. We performed multiple linear regression analysis using PWV, C-IMT, CAIx as dependent variables and the anthropometrics indices as predictors following three models. First model without adjustment. In a second model to include as adjustment variables age and gender (male = 1; female = 0), in the third model the presence of other cardiovascular risk factors (diabetes, hypertension, lipid lowering drugs, and smoking) and finally in the fourth model: systolic blood presure, total cholesterol, high sensitivity C-reactive protein, serum glucose, antidiabetic drugs and atherosclerotic plaques. The data were analyzed using the SPSS version 18.0 statistical package (SPSS Inc., Chicago, Illinois, USA). A value of P < 0.05 was considered statistically significant.

## Results

Table 1 shows the clinical and demographic characteristics of the 305 subjects included in the study (diabetics 32.8%, hypertensive subjects 37.0% and healthy individuals 30.2%), together with the anthropometric measures, inflammatory markers, central and peripheral arterial pressure, measures of arterial stiffness, subclinical atherosclerosis and wave reflection.

**Table 1:** General demographic and clinics characteristics

	Global n = 305	Healthy n = 92 (30.20)	Diabetic n = 100 (32.80)	Hypertensive n = 113 (37.00)	p
Age	53.37 ± 12.04	48.56 ± 11.85	59.29 ± 10.56	52.05 ± 11.33	< 0.001
Male (n. %)	163 (53.40)	34 (37.00)	65 (65.00)	64 (56.60)	< 0.001
Female (n.%)	142 (46.60)	58 (63.00)	35 (35.00)	49 (43.40)	< 0.001
Dyslipidemia (n. %)	121 (39.70)	12 (13.00)	73 (73.00)	36 (31.90)	< 0.001
Smoking (n. %)	71 (23.30)	24 (26.10)	20 (20.00)	27 (23.90)	0.597
BMI (Kg/m^2^)	27.92 ± 4.53	25.73 ± 3.49	29.90 ± 5.24	27.96 ± 3.75	< 0.001
Waist circumference (cm.)	95.80 ± 12.31	89.57 ± 9.53	102.32 ± 12.63	95.16 ± 11.25	< 0.001
Body fat percentage	34.06 ± 6.97	33.89 ± 7.04	34.67 ± 7.49	33.69 ± 6.47	0.589
Waist to height ratio	0.58 ± 0.08	0.54 ± 0.05	0.62 ± 0.09	0.57 ± 0.06	< 0.001
Total cholesterol (mg/dL)	205.36 ± 40.40	207.68 ± 38.70	189.81 ± 38.33	217.23 ± 39.41	< 0.001
HDL-cholesterol (mg/dL)	53.85 ± 13.70	58.10 ± 14.29	48.92 ± 11.13	54.82 ± 14.05	< 0.001
LDL-cholesterol (mg/dL)	126.28 ± 34.83	129.97 ± 34.31	111.19 ± 29.82	136.94 ± 35.01	< 0.001
Triglyceride (mg/dL)	104 (74-144.5)	80 (60.25-114.00)	118 (86.25-174.25)	109 (83.00-153.50)	< 0.001
Serum glucose (mg/dL)	89 (80-107.5)	82 (77.00-88.00)	130 (106.00-149.75)	85 (78.50-93.00)	< 0.001
hsCRP (mg/dL)	0.16 (0.09-0.33)	0.13 (0.06-0.23)	0.17 (0.09-0.47)	0.17 (0.09-0.29)	0.043
HOMA index	1.67 (0.98-3.02)	1.07 (0.70-1.75)	2.43 (1.49-4.24)	1.55 (1.06-2.75)	< 0.001
Fibrinogen (mg/dL)	320.52 ± 62.51	319.88 ± 60.11	328.77 ± 67.29	313.73 ± 59.62	0.224
Office SBP (mmHg)	133.61 ± 19.50	115.23 ± 14.21	137.10 ± 18.68	145.48 ± 11.37	< 0.001
Office DBP (mmHg)	84.21 ± 12.27	74.40 ± 8.92	83.10 ± 11.33	93.18 ± 8.33	< 0.001
Peripheral pulse pressure (mmHg)	49.40 ± 12.77	40.83 ± 8.93	54.00 ± 13.39	52.31 ± 11.46	< 0.001
Central SBP (mmHg)	125.28 ± 19.57	107.52 ± 13.69	128.86 ± 17.82	137.86 ± 12.95	< 0.001
Central DBP (mmHg)	84.87 ± 12.86	74.71 ± 9.41	84.31 ± 11.67	94.36 ± 8.87	< 0.001
Central Pulse pressure (mmHg)	40.41 ± 12.12	32.82 ± 8.48	44.55 ± 12.17	43.50 ± 11.78	< 0.001
PWV (m/s)	8.59 ± 2.29	7.03 ± 1.42	9.84 ± 2.35	8.80 ± 2.08	< 0.001
C-IMT mean (mm.)	0.71 ± 0.11	0.64 ± 0.09	0.77 ± 0.12	0.70 ± 0.10	< 0.001
C-IMT maximum (mm.)	0.88 ± 0.14	0.80 ± 0.11	0.96 ± 0.14	0.87 ± 0.12	< 0.001
CAIx	30.33 ± 12.07	29.27 ± 13.28	30.77 ± 11.33	30.89 ± 11.61	0.592
PAIx	91.51 ± 20.67	88.27 ± 19.74	94.73 ± 22.96	91.63 ± 19.11	0.109
ABI	1.11 ± 0.13	1.15 ± 0.11	1.09 ± 0.15	1.11 ± 0.11	0.002
Antihypertensive drugs (n. %)	72 (23.6)	0(0.0)	70 (70.0)	2 (1.8)	< 0.001
Antidiabetic drugs (n. %)	87 (28.5)	0(0.0)	87 (87.0)	0(0.0)	< 0.001
Lipid lowering drugs (n. %)	69 (22.6)	0(0.0)	58 (58.0)	11 (9.7)	< 0.001
Atherosclerotic plaques (n. %)	37 (12.1)	1 (1.1)	25 (25.0)	11 (9.7)	< 0.001

BMI: Body mass index. HDL: High-density lipoprotein. LDL: Low-density lipoprotein. hsCRP: High sensitivity c-reactive protein. SBP: Systolic blood pressure. DBP: Diastolic blood pressure. PWV: Pulse wave velocity. C-IMT:Intima-media thickness. CAIx: Central augmentation index. PAIx: Peripheral augmentation index. ABI: ankle-brachial index.

Data for qualitative variables are expressed as n (%) and quantitative variables as mean ± standard deviation or mean (interquartile range)

p: statistically significant differences (p < 0.05).

**Figure 1:**
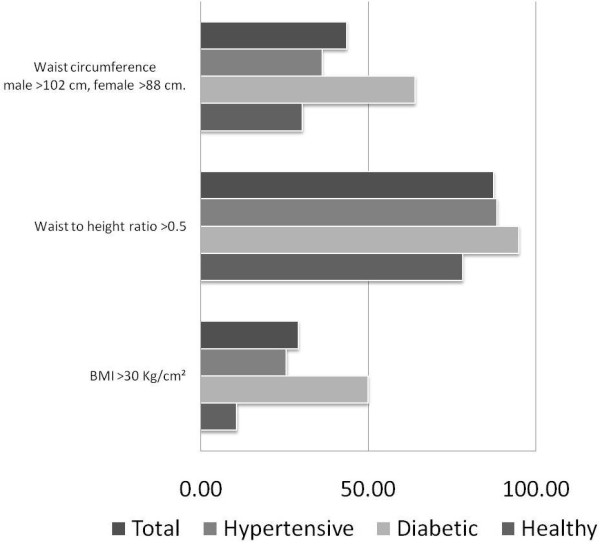
**Percentage of subjects in each group with body mass index greater than 30 Kg/m^2^, waist circumference greater than 102 cm in men and 88 cm in women and waist/height ratio greater than 0.5**.

The diabetics were older, with a higher percentage of dyslipidemia and a greater proportion of abdominal obesity and BMI > 30 than the hypertensive and healthy subjects (p < 0.01), while WHtR > 0.5 was similar to that recorded in the other groups (p = 0.066) (Figure 1).

The correlation between different anthropometric measures was high between BMI and the two abdominal obesity measures (WC and WHtR) (r = 0.858 and 0.877), moderate between BFP and BMI and WHtR, respectively (r = 0.506 and 0.538), and discrete between BFP and WC (r = 0.286).

The parameters assessing abdominal obesity (WC and WHtR) showed a positive correlation to PWV and C-IMT in the studied groups. BFP was positively correlated to the central augmentation index (CAIx) in healthy individuals, and to the central and peripheral augmentation indices in healthy and hypertensive subjects. No relationship between the arterial pressure values and anthropometric parameters was recorded in the hypertensive individuals. Lastly, a positive correlation was observed between inflammatory markers such as high sensitivity C-reactive protein (hs-CRP) and fibrinogen, and most of the anthropometric measures in diabetics or hypertensive individuals (Table 2).

**Table 2:** Pearson correlations between arterial stiffness and anthropometrics measurements

	**Healthy n = 92 (30.20)**					**Diabetic n = 100 (32.80)**				**Hypertensive n = 113 (37.00)**		
	
	BMI	WC	BFP	WHtR	BMI	WC	BFP	WHtR	BMI	WC	BFP	WHtR
Office SBP (mmHg)	0.114	0.285**	-0.257*	0.126	0.299**	0.379**	0.034	0.343**	0.009	0.016	-0.051	0.058
Office DBP (mmHg)	0.141	0.195	-0.151	0.112	0.457**	0.433**	0.142	0.409**	0.037	0.045	0.022	0.009
Peripheral pulse pressure (mmHg)	0.041	0.259*	-0.258*	0.089	0.031	0.163	-0.073	0.133	-0.018	-0.017	-0.070	0.052
Central SBP (mmHg)	0.068	0.187	-0.140	0.122	0.267*	0.322**	0.033	0.315**	-0.146	-0.156	-0.037	-0.041
Central DBP (mmHg)	0.211*	0.249*	-0.125	0.156	0.367**	0.350**	0.050	0.316**	0.022	0.032	0.044	0.021
Central Pulse pressure (mmHg)	-0.124	0.026	-0.087	0.024	0.038	0.136	0.001	0.158	-0.177	-0.195*	-0.079	-0.061
PWV (m/s)	0.078	0.209*	0.013	0.219*	0.230*	0.342**	0.163	0.357**	0.232*	0.267**	0.203*	0.335**
C-IMT mean (mm.)	0.093	0.228*	0.008	0.271**	0.053	0.229*	-0.074	0.176	0.223*	0.249**	0.101	0.306**
C-IMT maximum (mm.)	0.039	0.174	0.001	0.215*	0.051	0.231*	-0.072	0.177	0.257**	0.280**	0.108	0.325**
CAIx	-0.207*	-0.193	0.306**	0.054	-0.105	-0.150	0.063	-0.007	-0.284**	-0.285**	0.106	-0.083
PAIx	-0.215*	-0.172	0.300**	0.069	-0.057	-0.120	0.167	0.027	-0.115	-0.229*	0.263**	0.031
ABI	0.063	0.090	0.205	0.132	-0.114	-0.149	-0.075	-0.125	0.031	-0.060	-0.073	0.010
Fibrinogen (mg/dL)	0.172	-0.015	0.299**	0.211	0.337**	0.401**	0.287**	0.435**	0.202*	0.184	0.233*	0.257**
hsCRP (mg/dL)	0.306**	0.288**	0.180	0.301**	0.442**	0.442**	0.356**	0.475**	0.214*	0.266**	0.251*	0.316**
HOMA index	0.246	0.126	0.087	0.093	0.231*	0.303**	0.179	0.247*	0.427**	0.444**	0.108	0.465**

BMI: Body mass index. WC: Waist circumference. BFP: Body fat percentage. WHtR: Waist to height ratio SBP: Systolic blood pressure. DBP: Diastolic blood pressure. PWV: Pulse wave velocity. C-IMT: Carotid intima-media thickness. CAIx: Central augmentation index. PAIx: Peripheral augmentation index. ABI: ankle-brachial index. hsCRP: High sensitivity c-reactive protein. p: statistically significant differences * p < 0.05 ** p < 0.01

**Figure 2:**
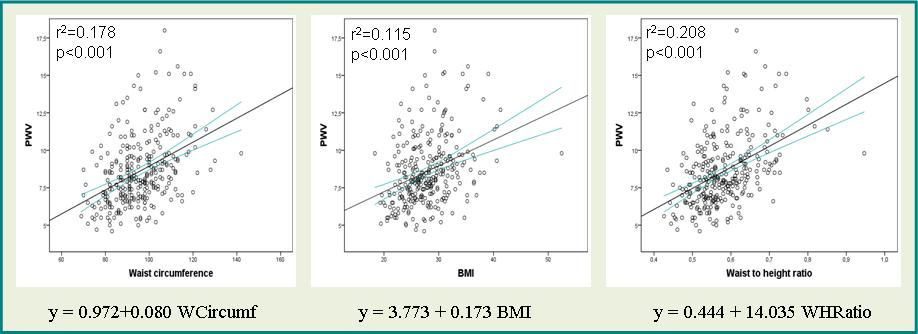
**PWV values plotted against anthropometric indices (WC, WHtR and BMI)**. The figures show regression line.

Figure 2 shows the simple regression straight line of PWV versus the anthropometric parameters.

After adjusting for age, gender and cardiovascular risk factors, the multiple regression analysis found that for every 0.1 point increase in WHtR, the PWV increased 0.041 m/sec and C-IMT increased 0.001 mm. Likewise, a 1 cm increase in WC implied an increase of 0.029 m/sec in PWV, 0.001 mm in C-IMT, and a decrease of 0.141 in CAIx. A 1 kg/m^2 ^increase in BMI in turn implied an increase of 0.052 m/sec in PWV, and a decrease of 0.270 in CAIx. Lastly, every 1% increase in BFP implied and a decrease of 0.284 in CAIx (Table 3).

**Table 3:** Models with PWV, C-IMT, and CAIx as dependent variables and anthropometric indices as predictors.

				PWV				C-IMT				CAIx	
		
		**R**^**2:**^	*β*	95% CI	p	**R**^**2:**^	*β*	95% CI	p	**R**^**2:**^	*β*	95% CI	p
	1st model	0.115	0.173	0.118 to 0.228	< 0.001	0.068	0.007	0.004 to 0.009	< 0.001	0.025	-0.439	-0.760 to -0.118	0.008
**BMI**	2^nd ^model	0.399	0.127	0.081 to 0.173	< 0.001	0.474	0.004	0.001 to 0.006	0.001	0.294	-0.434	-0.716 to -0.153	0.003
	3^rd ^model	0.453	0.091	0.044 to 0.138	< 0.001	0.504	0.002	< 0.001 to 0.005	0.036	0.304	-0.476	-0.777 to -0.176	0.002
	4^rd ^model	0.542	0.052	0.003 to 0.101	0.038	0.557	0.002	0.000 to 0.004	0.067	0.270	-0.493	-0.824 to -0.162	0.004
													
	1st model	0.178	0.080	0.060 to 0.099	< 0.001	0.144	0.004	0.003 to 0.004	< 0.001	0.030	-0.172	-0.286 to -0.057	0.003
**Waist circumference**	2^nd ^model	0.413	0.056	0.038 to 0.075	< 0.001	0.486	0.002	0.001 to 0.003	< 0.001	0.287	-0.154	-0.264 to -0.043	0.007
	3^rd ^model	0.470	0.045	0.026 to 0.063	< 0.001	0.511	0.001	< 0.001 to 0.002	0.003	0.298	-0.161	-0.278 to -0.044	0.007
	4^rd ^model	0.533	0.029	0.009 to 0.048	0.004	0.562	0.001	0.000 to 0.002	0.011	0.262	-0.141	-0.271 to -0.010	0.035
													
	1st model	0.019	0.043	0.007 to 0.080	0.019	0.001	< 0.001	-0.001 to 0.002	0.642	0.028	0.295	0.086 to 0.504	0.006
**% Fat**	2^nd ^model	0.368	0.070	0.033 to 0.107	< 0.001	0.441	0.001	-0.001 to 0.003	0.240	0.322	-0.322	-0.545 to -0.099	0.005
	3^rd ^model	0.443	0.055	0.019 to 0.090	0.003	0.483	0.001	-0.001 to 0.002	0.522	0.330	-0.320	-0.550 to -0.091	0.006
	4^rd ^model	0.527	0.033	-0.003 to 0.069	0.075	0.548	0.590	-0.001 to 0.002	0.954	0.286	-0.284	-0.540 to -0.028	0.030
													
	1st model	0.208	14.035	10.918 to 17.152	< 0.001	0.149	0.584	0.425 to 0.742	< 0.001	0.001	0.614	-18.967 to 20.195	0.951
**WHtR**	2^nd ^model	0.413	8.887	6.010 to 11.765	< 0.001	0.479	0.251	0.117 to 0.384	< 0.001	0.283	-21.317	-39.259 to -3.376	0.020
	3^rd ^model	0.466	6.915	3.941 to 9.889	< 0.001	0.506	0.170	0.029 to 0.311	0.018	0.292	-22.037	-41.479 to -3.135	0.023
	4^rd ^model	0.530	4.062	0.911 to 7.212	0.012	0.557	0.143	-0.003 to 0.290	0.050	0.259	-19.957	-41.377 to -1.463	0.068

PWV: Pulse wave velocity. C-IMT: Intima media thickness.CAIx: Central augmentation index. BMI: Body mass index. % Fat: body fat percentage. WHtR: Waist to height ratio.

p: statistically significant differences (p < 0.05).

First model: No adjusted.

Second model: Regression model constructed with BMI, waist circumference, body fat percentage and waist height ratio as predictors variables adjusted by gender and age.

Third model: Regression model constructed with BMI, waist circumference, body fat percentage and waist height ratio as predictors variables adjusted by gender, age, to be diabetic, to be hypertensive, lipid lowering drugs and smoking.

Fourth model: Regression model constructed with BMI, waist circumference, body fat percentage and waist height ratio as predictors variables adjusted by gender, age, to be diabetic, to be hypertensive, lipid lowering drugs, smoking, systolic blood pressure, total cholesterol, high sensitivity c-reactive protein, serum glucose, antidiabetic drugs and atherosclerotic plaques.

## Discussion

The present study shows that the measures of abdominal obesity (WHtR and WC) are better than the general obesity indicators (BMI and BFP) in predicting arterial stiffness as evaluated by PWV and subclinical atherosclerosis evaluated by C-IMT, independently of the presence of diabetes or hypertension. Therefore, WC and WHtR are the most useful parameters for estimating arterial stiffness in clinical practice.

A number of studies have analyzed the association between C-IMT and abdominal and general obesity parameters. Yan et al. [35] reported a good correlation between BMI and WC, though the waist-to-hip ratio was the parameter that best predicted C-IMT. In the study of Maher et al. [36], after adjusting for age, gender and the presence of cardiovascular risk factors, WHtR and BMI were seen to maintain their relationship to C-IMT. In both studies, and coinciding with the results of earlier publications [37], the measures that adjust WC were seen to be the best predictors of C-IMT. We did not measure the waist-to-hip-ratio, though another parameter that adjusts WC according to height was used, i.e., WHtR. However, in contrast to other studies, our findings were confirmed in all three patient groups considered (healthy individuals, diabetics and hypertensive subjects).

Likewise, we found a positive correlation between the anthropometric measures and PWV. Our results are in line with the observations of Ko et al. [23] and Wildman et al. [25]. Again, the behavior proved similar in all three groups studied.

Since the arterial stiffness is a surrogate marker between the appearance of risk factors and cardiovascular outcomes, our findings support the importance of anthropometric measures as a risk factors [38].

Maher et al. examined the relationship between the augmentation index and the anthropometric indices - only age being found to remain on performing the multivariate analysis. We did record a significant relationship, though of a negative sign. This may have been due to the fact that in our series the obese individuals (BMI > 30 kg/m^2^) presented a CAIx of 31.3 versus 28.0 in those with BMI < 30. In the case of BFP, the behavior was not uniform among the three groups. BFP was positively correlated to the CAIx in healthy individuals, and to the central and peripheral augmentation indices in healthy and hypertensive subjects - no such relationship being observed among diabetics.

In contrast to Tison et al. [39], we observed no relationship between the different anthropometric indices and ABI, and no consistent relationship with the arterial pressure measures (central and peripheral). This fact was more relevant in the hypertensive group - antihypertensive treatment being the most likely explanation.

The main limitation of our study is its cross-sectional design, which precludes the definition of causality and analysis of the behavior over time of the different stiffness parameters in relation to the different anthropometric indices considered. On the other hand, with a study design of this kind it would also be necessary to increase the sample size, since a larger number of individuals in each group (healthy, diabetic and hypertensive) could help clarify which anthropometric parameters are best related to arterial stiffness in each of them.

## Conclusions

Based on the results obtained in our study, it can be concluded that, independently of the presence of diabetes or hypertension, the measures of abdominal obesity (WHtR and WC) correlates better than BMI and BFP with arterial stiffness and subclinical atherosclerosis evaluated by PWV and C-IMT respectively. However, the association with wave reflection (evaluated by CAIx) was better with general adiposity measures.

## List of abbreviations

BMI: Body mass index; WC: Waist circumference; BFP: Body fat percentage; WHtR: Waist/height ratio; C-IMT: Intima-media thickness of the common carotid artery; PWV: Pulse wave velocity; CAIx: Central augmentation index; ABI: Ankle brachial index.

## Competing interests

The authors declare that they have no competing interests.

## Authors' contributions

JIR: devised the study, designed the protocol, participated in fund raising, interpretation of results, prepared the manuscript draft and corrected the final version of the manuscript. MAG and ER participated in the study design, interpretation of results, and manuscript review. CA participated in the study design, data collection and manuscript review. MCP performed all analytical methods, interpretation of results, and manuscript review. LG participated in the protocol design, fund raising, analysis of results, and final review of the manuscript. Finally, all authors reviewed and approved the final version of the manuscript.

## Pre-publication history

The pre-publication history for this paper can be accessed here:

http://www.biomedcentral.com/1471-2261/12/3/prepub
